# Implementation Barriers in Telesimulation as an Educational Strategy: An Interpretative Description

**DOI:** 10.7759/cureus.17852

**Published:** 2021-09-09

**Authors:** Samuel E Gutierrez-Barreto, Fernando D Argueta-Muñoz, Jessica D Ramirez-Arias, Emilio Scherer-Castanedo, Laura S Hernández-Gutiérrez, Hugo E Olvera-Cortés

**Affiliations:** 1 Department of Integration of Medical Sciences, National Autonomous University of Mexico, Mexico City, MEX

**Keywords:** interpretative description, telesimulation, undergraduate medical education, simulation training, qualitative research

## Abstract

Introduction

Telesimulation is one of the different methodologies for distance learning to promote competency in medical trainees. This methodology needs to have professors, students, and standardized patients in one session to perform a teleconsultation. Telesimulation could lead to multiple implementation barriers. This study aims to describe the implementation barriers through the perspective of the professors, students, and standardized patients in a telesimulation scenario in undergraduate medical education.

Method

We designed and applied a telesimulation scenario in undergraduate medical students. Then we conducted an online questionnaire with the critical incidents technique. The study sample was 18 professors, 26 standardized patients, and 407 students

Results

We describe a taxonomy with five categories and each one with different subcategories: knowledge (clinical simulation, theoretical over the clinical case, and use of simulators), facilities (access, time of use, and functionality), financing (payment to staff and purchase of equipment), attitude (acceptance and emotion), and participants (communication, collaborative work, and debriefing).

Conclusion

The description of the implementation barriers through multiple perspectives generates a taxonomy that could improve the quality of the telesimulation. This taxonomy is a proposal to consider the design, implementation, and evaluation when a telesimulation is implemented. The taxonomy could generate a structured plan when the educators implement the telesimulations at their own institutions considering all the barriers proposed.

## Introduction

Educational strategies have evolved since the development of information technologies that use virtual and face-to-face environments for education. According to competency-based education, students develop adequate skills, aptitudes, and abilities to exercise their profession [1]. However, in virtual environments is challenging to developing educational strategies and achieve mastery of technical and social skills [2].

The COVID-19 pandemic has transformed medical education due to social distancing measures hindering face-to-face training. Different methodologies for distance learning have been adapted, including clinical simulation [3]. Among the virtual educational strategies that promote the development of skills and aptitudes in the health professional is telesimulation [4]. This strategy was carried out more than a decade ago and is defined as the implementation of a simulation at a physical distance from the participants [5,6]. Around the world, different simulation centers use telesimulation as an educational strategy for the training of health professionals [7,8].

In recent months, this strategy has been forced to evolve, incorporating new telecommunication tools that allow regular sessions, with large capacity and topics suitable for comprehensive medical development, and has proven to be comparable with its face-to-face version [8,9]. To carry out telesimulation, the facilitator requires an understanding of experiential learning theory, which is helpful for the development of the cognitive and affective domains of the student [4]. However, telesimulation still has advantages and disadvantages that need to be evaluated and understood [8].

Implementation barriers in face-to-face simulation have been described as the following: physical interaction, human resources (sufficient and trained), materials (specialized facilities, simulators, and specific computer equipment), attitude to the simulation strategy, knowledge and skills of the participants to carry out the scenario, financing, and having evidence of the importance of simulation for education [10,11]. Only the communication between students and facilitators has been described as a barrier to the implementation of telesimulation [12].

There is little information about the barriers to deploying simulation in virtual environments. A taxonomy of the implementation barriers of virtual simulation would allow telesimulations with the fewest possible incidents. This study aims to identify and describe the implementation barriers through the perspective of the professors, students, and standardized patients in a telesimulation scenario in undergraduate medical education.

## Materials and methods

We designed and applied the telesimulation as described in Table 1. Then we used interpretative descriptions. This method assumes that human interactions are contextualized and that the people studied have a deep understanding of the human phenomenon and how its components interact [13]. In this study, the technique of critical incidents was used to collect information through an online questionnaire through a Google Form™ (Google, Mountain View, California) [14]. 

**Table 1:** Telesimulation description

Element	Description
Instructional design	The session had a three-stage structure, briefing (10min), simulated scenario (20min), and debriefing (30min). Experts in the area were invited to design and validate the telesimulation scenario. The coordinators of the simulation ensure the standardization of the activity.
Participant orientation	The coordinators send an email invitation with information about the simulation scenario, logistics, instructions, and session objectives. The instructors allocated the first ten minutes of the session to establish a safe learning environment, to resolve doubts about the learning objectives and the duration of the scenario.
Simulator type	The simulation required the participation of a Standardized Patient (SP).
Simulation environment	Environment simulation used a video conference platform; all participants were at home. The instructors generated the atmosphere with a virtual background of a doctor's office and clothing according to their role to achieve realism.
Simulation event/scenario	The scenario consisted of a follow-up prenatal control visit to a 26-year-old patient with 28 weeks gestation, who presented headache, tinnitus, nausea, and phosphenes of two days of evolution. The simulation event took place in a conference with three students, one SP, one nursing staff, and one professor. The learning objectives were: (1) establish the differential diagnosis of pregnancy complications; (2) classify hypertensive disease of pregnancy according to its clinical characteristics; and (3) promptly refer the patient to the next level of care.
Feedback	The feedback consisted of a reflective process called debriefing with good judgment. The professor and the SP guide the debriefing.

The first screen of the questionnaire showed the study’s objective and informed consent, to continue its acceptance was required. Then we asked for sociodemographic information (sex, age, and experience in an online simulation). Subsequently, through a series of structured open answered questions, the description of the experience was ensured. All students (S), professors (P), and Standardized Patients (SP) in the curriculum of the Faculty of Medicine of the National Autonomous University of Mexico in September 2020 who participated in the telesimulation were invited. All the authors analyzed the collected testimonies inductively, comparatively, and iteratively. We asked each participant to accept informed consent online before the questionnaire’s response. The Ethics Committee of the University agreed that we could proceed as a quality assurance project and provided the appropriate permits to carry out in alignment with the declaration of Helsinki.

We performed a structured review of the literature in the following databases: Cumulative Index to Nursing and Allied Health Literature (CINAHL), Education Resources Information Center (ERIC), PsychInfo, ProQuest, Francis, Scopus, EBSCO, and Pubmed to construct a category scheme. In each database, the following terms were used: simulation, implementation barriers, medical education, and clinical education with the filters: title, abstract, and from January 2005 to January 2021 in English and Spanish. Testimonials related to some categories were collected in a spreadsheet specifying the role played during telesimulation: P, S, and SP. Each critical incident was coded using a line-by-line approach, and a code was assigned depending on the conceptual domain. Constant comparison was performed to redefine the coding and ensure that the authors’ concepts were consistent. During this stage, the authors modified the main category through an iterative process until all the authors decided that no further changes could be generated. During the coding, some testimonies were classified into two categories; for this, the authors Gutierrez-Barreto and Olvera-Cortés identified the relevant topic to simplify the testimonies.

## Results

During one week of telesimulation scenarios, we obtained: 407 out of 509 (79%) testimonies for S, 26 out of 30 (87%) for SP, and 18 out of 51 (35%) for P. In the consensus performed, we construct a taxonomy of implementation barriers (Table 2). In the next paragraphs, the main domains of the taxonomy are described, exemplified, and discussed.

**Table 2:** Taxonomy of implementation barriers in telesimulation

Category	Subcategory	Definition
1. Knowledge	1.1 Clinical simulation	Participants have information about their role in the simulation method (pre-briefing, scenario, debriefing) and the scope of this.
	1.2 Theoretical over the clinical case	Participants use the data provided before, during, and after the scenario consistently to achieve the simulation objectives.
	1.3 Use of simulators	Skills that allow the correct preparation and use of the infrastructure of a scenario (face-to-face or remote) by the participant. It includes being responsible for the integration, maintenance, and management of the technology necessary to achieve the objective of the activity.
2. Facilities	2.1 Access	Available and affordable space to perform the simulation scenario physically or virtually at the right time.
	2.2 Time of use	Amount of time spent on simulation scenarios and their logistics in the case of multiple groups.
	2.3 Functionality	The space allocated should be sufficient and suitable for the distribution of participants, facilitators, and the type of activity.
3. Financing	3.1 Payment to staff	Economic stimulus to anyone involved in the design and implementation of the scenarios.
	3.2 Purchase of equipment	Acquisition of simulators, upgrades, consumables, and licenses.
4. Attitude	4.1 Acceptance	Consideration of simulation participants as a useful educational strategy.
	4.2 Emotion	Reactions of the participants and their recognition unrelated to an element in the simulation.
5. Participants	5.1 Communication	Verbal and nonverbal interactions between participants.
	5.2 Collaborative work	Distribution of tasks/roles to be performed.
	5.3 Debriefer	Perspective on the result obtained by the performance of the debriefer.

Knowledge

The barrier of knowledge understood as not having information about the simulation and its components was described in three sections: clinical simulation, case theory, and the use of simulators. The following testimonies exemplify the different perspectives.

"… I didn't know what I could ask for and what I couldn't. Whether it was right or possible to ask for a scan or talk to the patient while watching her studies so that there would be no uncomfortable silence." (S)

"... sometimes students do not know where the scenario takes place (an office, a health center, a hospital) or if the patient needs to be referred to continue treating him." (P)

"It was stressful because I thought I mastered the subject, but at the time of simulating the scenario, I realized that there were aspects that I needed to review or that I had skipped, so I didn’t know what to do." (S)

"We had already solved the case in a virtual classroom with multimedia and simulation of a similar case. But the moment the students must interrogate a patient, they can’t generate clinical data by themself, they get blocked and begin to interrogate disorderly..." (SP)

"One of the obstacles is the characterization by the age of the patient, but I think it is solved well with the moulage… I have little trouble with the virtual background, I will improve that point, to have the same atmosphere with the nurse and the patient." (P)

Facilities

This category considers the space allocated for the realization of the practice, whether face-to-face or virtual, and contemplates three fundamental sections: access, available time, and finally, if it is enough to work within it. The testimonies collected are presented below:

"The internet was our main problem. The communication was interrupted, and I was taken out of the session, so I missed 8 minutes." (S)

"Students generally have the habit of looking for information online; however, with the little time they have to solve the case, and if they are not well oriented with the patient, that ability is not very effective." (P)

"A student got stressed out due to connection problems including audio and video, so I asked for help to support, but it didn’t work, so I decided to keep contact by chat, to make her feel less stressed." (SP)

"Time is short, and therefore you have to organize it very well; there are connection failures, and it is understandable. I think it was a good experience considering it was the first one. There could be more organization, to start sessions faster." (S)

“The use of Zoom and the dynamics seemed excellent to me. The experience works as well as we were face-to-face because otherwise, we wouldn’t have had enough time to see the case deeply, and everything would have been verbal.” (S)

Financing

The financing for the implementation of simulation programs refers to any economic element necessary to carry out a learning activity with simulation, from the acquisition of consumables, software, simulators, and facilities to the payment to all the personnel involved. In the participants' commentaries, we did not identify concepts that could be categorized in this area.

Attitude

This category addresses the attitudinal barrier as one that by absolute subjectivity modifies the participant's performance during the simulation. This barrier can be understood from two perspectives. The first perspective is the acceptance of the simulation as a valuable educational strategy for their formation. The second is the emotional component of the participants and their recognition, without this being related to an element of the simulation itself. Below are some testimonies that exemplify this category from different perspectives.

"I think I didn’t take advantage of the case achieved because telemedicine benefits patients a lot, but as a teaching practice, I didn't feel so." (S)

"Well, my experience is excellent; I think it is of great help for the students to know how to interrogate and have a good patient-doctor relationship..." (SP)

"The students resented at first not working with a patient in physical, but using the telesimulation resource is super important and brought them pretty close to this new reality as a tool." (P)

"... the degree of dispersion is very high. The standardized patient had to use more lifesavers, which would allow the student to be mobilized more and taken out of his daze." (P)

"... the facilitators behaved very well with us, helped us a lot and gave us a lot of confidence to perform the activity" (S)

 

Participants

This final category refers to the interactions between the participants of the simulation and also the communication, teamwork, and the perception of the debriefer's performance.

"I feel that my verbal and non-verbal language was adequate, although it gives me a bit of work to know when to intervene" (SP)

"The students resented at first not working with a patient in physical, and even the part in working in a remote team for the taking of elections was hard to them." (P)

“Direct communication to others was not possible due to the fact of interruption when somebody else is speaking.” (S)

"Organizing with my peers is more complicated." (S)

"... I learned that teamwork is critical as well as to know how to give a teleconsultation in these times." (S)

"... the debriefing was very good, both for how my partner handled it, as well as the very active participation of the students." (SP)

"... rather than debriefing, they provided a feedback to the actions taken by the students, due to the deficits perceived." (P)

## Discussion

Knowledge

The participants’ lack of knowledge of telesimulation could be a limitation for the development and achievement of the objectives. To mitigate this barrier, those responsible for developing the scenario could verify the simulation design structure, mainly by conducting a pre-briefing with the characteristics recommended by the International Nursing Association for Clinical Simulation and Learning (INACSL) and the training of staff as standardized patients and facilitators [15]. All simulation participants need to be aware of how the infrastructure works for the performance of their role during the scenario. We recommend performing a simulation model for the participants and their expected objective according to their role and other skills that contribute to the realism of the scenario.

Facilities

Due to distance, size, the number of people, or distribution, a space with difficult access can make learning difficult and might cause frustration to the student and the simulation facilitator [16]. The testimonies show that access to a network and computer equipment that allow a stable connection and therefore a fluid development of the practice are determining factors when accessing telesimulation. Unfortunately, the only way to improve these resources lies within the student. Correct planning allows the student to prepare for any failure. Also, this planning should consider the time to reach the learning goal [17]. A period of time must be determined that allows the student to function without the pressure of a short limit or with plenty of time that may cause disinterest in the student. The scenario must end when no further progress can be made, whatever the cause, or when the aptitude to solve it has been demonstrated. This barrier is affected inversely proportionally by the difficulty of access to the practice and is a determinant for students’ performance.

The time of the scenario is sensible to the number of students in each simulation. This can be attributable to two factors: the first is the students’ ability for teamwork organization, and the second is in the debriefing session because it can be challenging to address all the aspects of each student.

Financing

In the face-to-face simulation, multiple authors refer to it as a barrier to implementation [18,19]. We consider that the participants did not direct it due to not being aware of the expenses involved in carrying out a telesimulation scenario, including the acquisition of licenses for videoconferencing platforms, internet, cameras, among others that without them a telesimulation could not be carried out.

Attitude

Telesimulation is a relatively new methodology [4], in this case, evokes an equally unpopular medical area, telemedicine [20]. So, the use of these in medical education seems of little use to most novice students because of their little approach with both techniques throughout their training. One possible solution is the diversification of undergraduate medical education for the inclusion of these methodologies. The telesimulation highlights the already known fact that emotion management can hinder the development of the scenario or the achievement of the proposed learning objectives [21]. Emotions can become one more element that contributes to the participants’ learning if there is a facilitator trained for this purpose, creating a safe analysis environment and providing the necessary support to channel that emotion in favor of problem-solving.

Participants

To detect barriers in distance communication is essential to know the experience and opinions of those involved in the communication process [22]. In this section, the participants agree that communication through a virtual medium becomes an obstacle since it hinders decision-making and collaborative work among those who participate in the simulation, especially among students. The communication barrier within the simulation had not been described as telesimulation is a little-used methodology. The fact that all participants have been affected and that it is detrimental to making decisions and appropriate collaborative work demonstrates the need to find solutions to this problem. One of the ways to solve the problem of communication, active participation, and collaborative work is to train participants in the use of the platform and encourage students to assign roles to distribute the tasks they propose [22]. During the debriefer performance, we find that the professors' and students' perceptions contrast when evaluating the debriefing quality. While professors found areas for improvement that involved their effectiveness, students perceived that debriefing had been helpful and would help them in the future. This distinction may be due to academic or professional experience and what professors consider to be of greater relevance than the student's inexperience.

In a recent study, 12 tips for conducting telesimulation were presented [23]. In contradistinction, we described 13 possible barriers to consider during the implementation of telesimulation in undergraduate medical education. The addition of this analysis is the brief definitions of these barriers, taking different perspectives into account. This taxonomy could be used in both telesimulation and face-to-face environments. Although, it is crucial to implement it in a simulation design model to give practical validation. One of the limitations of this study is that the participants were selected in a specific simulation center which may cause bias in the perception of these barriers. Another limitation is that only participants were included; we suggest further studies including simulation center managers and multiple simulation centers to obtain different perspectives of the barriers deployed. Also, to generate an assessment form to evaluate the telesimulation design.

## Conclusions

The description of the implementation barriers through multiple perspectives generates a taxonomy that could improve the quality of telesimulation. This taxonomy is a proposal to consider the design, implementation, and evaluation when a telesimulation is implemented. The taxonomy could generate a structured plan when the educators implement the telesimulations at their institutions, considering all the barriers proposed.
